# The Tactile Window to Consciousness is Characterized by Frequency-Specific Integration and Segregation of the Primary Somatosensory Cortex

**DOI:** 10.1038/srep20805

**Published:** 2016-02-11

**Authors:** Julia Natascha Frey, Philipp Ruhnau, Sabine Leske, Markus Siegel, Christoph Braun, Nathan Weisz

**Affiliations:** 1 CIMeC, University of Trento, via delle Regole, 101, 38123 Mattarello (TN), Italy; 2 Center for Cognitive Neuroscience, Paris-Lodron Universität Salzburg, Hellbrunnerstr. 34, 5020 Salzburg, Austria; 3 Department of Psychology, University of Konstanz, Universitätsstr. 10, 78464 Konstanz, Germany; 4 Centre for Integrative Neuroscience & MEG Center, University of Tübingen, Otfried-Müller-Str. 47, 72076 Tübingen, Germany

## Abstract

We recently proposed that besides levels of local cortical excitability, also distinct pre-stimulus network states (windows to consciousness) determine whether a near-threshold stimulus will be consciously perceived. In the present magnetoencephalography study, we scrutinised these pre-stimulus network states with a focus on the primary somatosensory cortex. For this purpose participants performed a simple near-threshold tactile detection task. Confirming previous studies, we found reduced alpha and beta power in the somatosensory region contralateral to stimulation prior to correct stimulus detection as compared to undetected stimuli, and stronger event-related responses following successful stimulus detection. As expected, using graph theoretical measures, we also observed modulated pre-stimulus network level integration. Specifically, the right primary somatosensory cortex contralateral to stimulation showed an increased integration in the theta band, and additionally, a decreased integration in the beta band. Overall, these results underline the importance of network states for enabling conscious perception. Moreover, they indicate that also a reduction of irrelevant functional connections contributes to the window to consciousness by tuning pre-stimulus pathways of information flow.

Studies investigating pre-stimulus effects in near-threshold (NT) paradigms observed that correctly perceived stimuli are preceded by low alpha power in task-relevant areas. This was shown for the visual^1^,^2^,^3^,^4^ and the somatosensory cortex^5^,^6^,^7^,^8^,^9^ depending on the task. These observations are usually interpreted according to the notion that alpha activity reflects the cortical excitability with strong alpha reflecting functional inhibition^10^,^11^. The straightforward rationale, thus, states that an upcoming NT stimulus will become conscious when pre-stimulus local excitability (e.g., in the visual cortex) is high such that a weak input causes ignition of relevant neural assemblies. Hence, alpha power effects are interpreted in local terms. Despite providing an intuitive explanation of pre-stimulus determinants of conscious perception that are well linked to a strong conceptual framework, this interpretation has a major shortcoming. As this viewpoint emphasizes local pre-stimulus cortical excitability, it predicts that successful stimulus detection depends on a bottom-up input sweep. In this case, effects in sensory regions should become evident immediately, as soon as an ignition threshold is crossed. Interestingly, evidence for this implicit prediction is rather scarce. In contrast, effects in sensory regions are reported to appear relatively late, probably due to recurrent activation from downstream areas^12^,^13^.

Apart from this empirical discrepancy of what should be expected if pre-stimulus effects were interpreted along the functional inhibition hypothesis, major neuroscientific frameworks of conscious perception stress a network perspective. For example the global neuronal workspace model (GNW)^14^ suggests that sensory stimuli only become conscious, if they are *globally* available for further cognitive processing. Recently, we have shown that conscious perception of a weak tactile stimulus is characterised by a stronger network integration of the secondary somatosensory cortex (SII) already in the pre-stimulus period^9^. In an accompanying framework called Windows to Consciousness (win2con)^9^,^15^, we have argued that this enhanced pre-stimulus integration reflects pre-established pathways of information flow, facilitating a more efficient stimulus-related spread of information throughout a distributed network. By extending the straightforward interpretation of pre-stimulus effects in terms of local cortical excitability, our framework adds a conceptual contribution to bridge the gap between the pre- and post-stimulus divide (i.e. what would be predicted based on a local excitability hypothesis, and what is actually observed in the post-stimulus period). Specifically, it does not predict early activation in sensory regions to determine conscious perception (for detailed argumentation, see^15^).

In our framework^9^,^15^, we have specifically emphasized *increased* pre-stimulus integration reflecting pre-established pathways of information flow. We have argued that increased coupling and enhanced network integration make stimulus processing more efficient, and thus more likely to be consciously perceived^9^,^16^. Stimulus processing, however, will also be optimized, if pre-stimulus functional connectivity is tuned to incoming sensory stimuli by *reducing irrelevant* connections. For instance, if task-relevant sensory areas (e.g., the primary somatosensory cortex in a unimodal tactile task) are strongly coupled with task-irrelevant areas (e.g., other sensory regions), processing of an incoming tactile stimulus could be impaired. Thus, it is likely that the tactile window to consciousness is not only characterized by improved coupling between sensory regions and task-relevant higher-order areas, but also by decoupling between sensory regions and task-irrelevant cortical areas.

In our pervious study^9^, we focused mainly on SII and its functional connectivity predisposing stimulus detection. Whereas sustained stimulus-related activity in SII has been shown to be essential for conscious perception^17^,^18^, a causal modelling study emphasized recurrent activity between SI and SII to underlie somatosensory awareness^19^. Most likely, stimulus-related activity in SI is necessary but not sufficient for conscious perception, whereas sustained activity in SII and recurrent processing between SI, SII, and higher-order areas form coalitions of neurons^20^, resulting in a conscious percept. Activity in SI and SII also show clear differences in other paradigms, such as somatosensory-motor regulation^21^, spatial attention^22^, change detection in multisensory environments^21^, and sustained electrical stimulation^23^. The exact roles, however, of SI and SII in the window to consciousness have not been explored so far, particularly regarding pre-established functional pathways.

Going beyond our previous study^9^, we investigated whether there are inverse pre-stimulus network patterns predisposing conscious perception as described above, with a particular focus on SI. To this end, we conducted a magnetoencephalography (MEG) study using a simpler NT detection task, in which participants were presented on their left index finger with a tactile stimulation (Fig. 1A). After each stimulus, they were prompted to indicate whether they had felt the stimulus or not. Then, we computed pre-stimulus time-frequency resolved global and local graph theoretical measures. In addition to previously described power decreases in the alpha and beta band in sensory areas prior to conscious perception, here we report for the first time simultaneous functional integration and segregation patterns for the relevant sensory areas. Specifically, we found increased pre-stimulus integration of the SI contralateral to stimulation in the theta band (increased local efficiency and local degrees), and decreased integration in the beta band (decreased local efficiency) prior to conscious perception. Overall, these findings have far-reaching consequences in understanding the prerequisites^24^ of conscious perception.

## Results

### Behaviour

Across all participants (N = 19), detection rate was 94% (SD: 5%) for catch trials, and 50% (SD: 9%) for experimental trials. The mean false alarm rate in sham trials was 7.5% (SD: 8.5%). Detection rate of experimental trials significantly differed from those of catch trials (T_1,18_ = −20.7, p < 0.001), but not from chance (T_1,18_ = 0.087, p = 0.93), indicating that stimulation at NT intensities was successful and participants were highly compliant.

### Post-stimulus event-related neural activity

**Figure 1:**
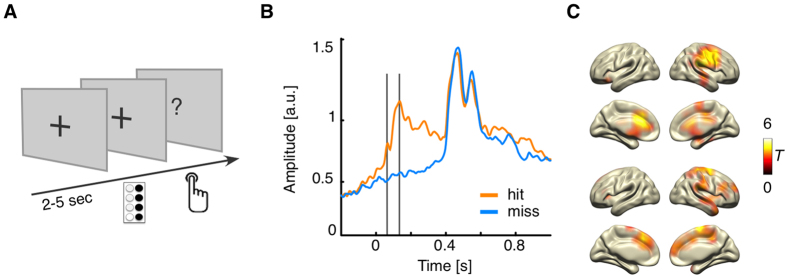
Design and event-related responses. (**A**) After a variable inter-trial interval between 2–5 s during which participants fixated a central cross, a tactile stimulus was presented on the tip of their left index finger for 50 ms at individual perceptual intensity. After 250 ms, stimulus presentation was followed by an on-screen question, and participants indicated their perception by pressing one of two buttons (‘detected’ or ‘undetected’). (**B**) Sensor-level event-related global field power in the detected (orange) and undetected (blue) condition for the gradiometer data. Marked with grey lines: First peak at 63 ms, second peak at 133 ms. (**C**) Source reconstruction of the two main sensor-level peaks marked in B at 63 ms (top) and 133 ms (bottom) for the contrast detected vs. undetected trials, masked at p_cluster_ < 0.05.

Sensor-level event-related magnetic fields (ERF) clearly show that stimuli reported as detected resulted in pronounced post-stimulus neuronal activity, whereas unreported stimuli did not (see Fig. 1B, C). The ERFs of both magnetometers and gradiometers were significantly different from around 0–500 ms and 0–550 ms post-stimulus, respectively, showing a first peak at 63 ms and a second peak at 133 ms (Fig. 1B). The first peak was relatively higher for the magnetometers (data not shown), suggesting a deeper source. This assumption was confirmed by reconstructing the sources of both peaks (Fig. 1C). Whereas the first evoked peak was localised to the somatosensory and motor areas contralateral to stimulation and the bilateral anterior cingulate gyrus, the second peak originated mainly in the primary and secondary cortices (SI and SII) contralateral to stimulation, and the bilateral superior frontal gyrus.

### Pre-stimulus power effects

The main statistical contrast of detected vs. undetected trials (see Statistical Testing) resulted in a significant negative cluster in both sensor types in the alpha band (gradiometers: p_cluster_ = 0.032, magnetometers: p_cluster_ = 0.034; data not shown), but not in the theta or beta band. The alpha effect seen in the peak magnetometer (MEG1311) lasted from around 700–100 ms pre-stimulus and was maximal at 11 Hz and 300 ms pre-stimulus. In contrast, the effect seen in the peak combined gradiometer (MEG1112 + 1113) emerged earlier lasting from 800–100 ms pre-stimulus, and was maximal at 11 Hz and 600 ms pre-stimulus. On a descriptive level, the topographies of the alpha effect in both sensor types were lateralised contralateral to the stimulation, and strongest in frontocentral sensors.

**Figure 2:**
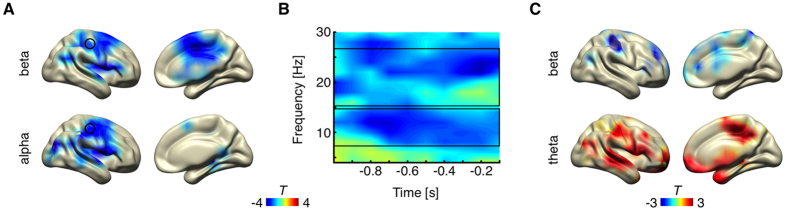
Pre-stimulus source-level power and functional connectivity. (**A**) Source reconstruction of the alpha (lower panel) and beta (upper panel) power effect (p_cluster_ < 0.05). Marked with a black circle is the voxel in SI with the maximal power effect in both frequency bands. (**B**) The time-frequency representation of the voxel with the maximal power effects marked with a black circle in A. Marked with black rectangles are the time-frequency windows that were used for the source-level power analysis (alpha: 8–14 Hz; beta: 16–26 Hz). (**C**) Local efficiency effect in the beta band (top; 16-26Hz) and in the theta band (bottom; 2-6Hz). For illustration purpose, the whole brain map is shown at p_uncorrected_ < 0.05.

On source-level, the statistical analysis of spectral power resulted in significant relative alpha and beta band power decreases prior to stimulus detection (p_cluster_ = 0.016 and 0.01, respectively). No power modulations were found in the theta band. Both effects were distributed across the post- and precentral gyri (BA3 and BA4) contralateral to stimulation, while the beta band effect also included the middle cingulate cortex (see Fig. 2A) contralateral to stimulation. The time-frequency representation for the voxel with the maximal effect in both frequency bands reveals that the alpha band effect lasts from 900–200 ms pre-stimulus with a peak from 800–700 ms pre-stimulus at 12 Hz (see Fig. 2B). Whereas the beta power effect also emerges around 900 ms pre-stimulus, it becomes strongest shortly before stimulus presentation (after around 400 ms pre-stimulus; see Fig. 2B).

### Pre-stimulus functional connectivity

To investigate the functional network architecture in the time window of interest (1000-100 ms pre-stimulus), we compared global and local graph theoretical measures (detected vs. undetected). There was no significant effect in any of the global graph theoretical measures (density, path length, efficiency, small-worldedness). Concerning the local metrics, we found significant effects for local degrees and efficiency in the right SI (contralateral to stimulation), but not for local clustering and betweenness. Local node degree and efficiency tested in the SI contralateral to stimulation were relatively increased prior to stimulus detection in the theta band (p_FDR_ < 0.05; see Fig. 2C). These effects were mainly driven by relative increased values at 6 Hz throughout the whole pre-stimulus period. In contrast, local efficiency in the contralateral SI was relatively decreased prior to conscious perception in the beta band (p_FDR_ < 0.05; see Fig. 2C), which was mainly driven by negative values from 16–18 Hz and 24–26 Hz between 900-600 ms pre-stimulus (data not shown). For illustration purposes, Fig. 2C shows the whole brain map of both effects (local efficiency in the beta and theta band), masked at p_uncorrected_ < 0.05).

## Discussion

In the present MEG study, we set out to investigate the pre-stimulus network integration of the task-relevant sensory area that predisposes conscious perception. First of all, we were able to replicate the well-established pre-stimulus alpha power modulations in task-relevant areas^2^,^4^,^6^,^9^. In addition to the alpha power effect, we provide more support for our framework win2con, by demonstrating that conscious tactile perception is indeed preceded by a modulated functional integration of task-relevant regions into the network. Specifically, analysis in the SI contralateral to stimulation revealed enhanced network measures in the theta band, and diminished network measures in the beta band. We interpret this integration/segregation pattern as a tuning of pre-established functional pathways to the relevant sensory inputs. As stated in the win2con framework^15^, this tuning of the functional architecture optimizes stimulus processing and predisposes conscious perception.

### Pre-stimulus alpha power in the SI contralateral to stimulation precedes conscious tactile perception

In the present study, we replicate previous findings concerning alpha power in task-relevant areas. Contrasting detected with undetected trials revealed relatively decreased alpha power in SI and SII, and distributed regions contralateral to stimulation. Oscillatory alpha activity is thought to reflect the cortical excitability state^10^,^11^. Thus, the relatively decreased alpha power prior to successful stimulus detection presented here can be interpreted as a release of functional inhibition of task-relevant areas, making stimulus processing more efficient and conscious perception more likely. This finding is in line with previous research regarding conscious tactile perception. Similar pre-stimulus alpha power modulations over posterior regions and the somatosensory cortices were shown to precede conscious somatosensory perception^6^,^9^. Regarding other modalities, pre-stimulus alpha power modulations inversely related to conscious perception were also shown, for instance, in vision^4^,^25^,^26^.

### Local efficiency modulations in the theta and beta band prior to conscious perception reflect integration and segregation of the SI contralateral to stimulation

Regarding the pre-stimulus functional connectivity, we set out to scrutinise our framework win2con^9^,^15^, which predicts that enhanced integration of task-relevant sensory areas within the whole brain network determines upcoming conscious perception. In line with our framework, we expected to find more efficient integration of such regions prior to correctly detected stimuli. In the present study, we focused on SI contralateral to stimulation, and analysed network dynamics using a graph theoretical approach^27^,^28^.

Using graph theoretical measures, we found higher pre-stimulus local efficiency and node degree values in the theta band in the SI contralateral to stimulation. Local node degree reflects the number of direct connections of a node, whereas local efficiency reflects the average path length between all directly connected nodes. Thus, these two measures strongly suggest that the contralateral SI is stronger and better integrated in the overall network. Enhanced integration of SI predisposes the propagation of stimulus-related activity to higher-order areas. This propagation causes the functional network to ignite, and the stimulus to be globally accessible, resulting in a conscious percept^12^,^29^. In contrast to our previous report^9^, in which we reported connectivity effects in the alpha band originating from SII, here we specifically focused on SI contralateral to stimulation. To do so, we employed a simpler tactile detection task, emphasizing the functional role of SI. This change in paradigm could also account for the differences in global graph theoretical measures. Whereas we have previously reported smallworldedness and global efficiency effects^9^, no such differences were observed in the present study. Overall, enhanced integration of the SI contralateral to stimulation in the theta band reflects pre-established functional pathways prior to conscious perception, which represent crucial components of a tactile prerequisite of consciousness.

In addition to the increased local node degree and efficiency effects in the theta band described above, the graph theoretical analysis revealed relatively decreased pre-stimulus local efficiency in the beta band in the same area. Even though, at first glance, this finding seems to disagree with the win2con framework^9^,^15^, it not only integrates well with our hypotheses but also adds important insights. The win2con framework states that pre-established functional pathways determine conscious perception by guiding information flow. Specifically, we have argued that pre-established functional pathways increase the likelihood that a weak sensory stimulus crosses the perceptual threshold and stimulus-related neural activity propagates to the global neuronal workspace^14^,^30^. Intuitively, our hypothesis seems to refer only to ‘more’ or ‘better’ pre-established functional pathways. However, stimulus processing is also arguably enhanced if pre-stimulus functional pathways are tuned to the processing of the incoming task-relevant sensory stimulus by *reducing* pathways that are detrimental to stimulus processing. Such irrelevant pathways could mean a) functional pathways between regions of no interest (spatial domain), or b) functional pathways occurring in an irrelevant time-frequency window (temporospectral domain). In either case, the reduction of irrelevant functional pathways benefits the processing of weak sensory stimuli by increasing the likelihood that the stimulus-related activity is propagated to relevant brain regions (for a similar argument for alpha power, see^11^,^31^. Thus, the observed segregation of the SI contralateral to stimulation is seen as a tuning mechanism. Accordingly, the relative decreases in local efficiency in the beta band reported in this study are reflecting an essential part of the win2con framework.

The use of graph theory is a promising approach to analyse functional neural networks, particularly during cognitive tasks. For instance, recent studies have reported changes in graph theoretical metrics during an active working memory task^32^, mathematical processing^33^, attention deployment^34^, and perception^9^,^16^,^35^. In particular, based on a visual oddball task, increased clustering, decreased modularity, and increased rich-club coefficients due to stronger interactions between hub-like nodes were proposed as the functional ‘fingerprint’ of cognition^35^. Similarly, decreased modularity after stimulus presentation was associated with awareness^36^. These findings are partly in line with the win2con framework, as they posit increased functional integration (increased clustering, or decreased modularity) to underlie conscious perception and cognition. Along these lines, the current study resulted in increased local efficiency in the theta band, reflecting a more efficient integration of SI in the system. However, the present findings also demonstrate how a frequency-dependent tuning of the network – in particular beta-band decreases in local efficiency in SI – is beneficial for perceptual performance.

Taken together, the increased theta band efficiency and the decreased beta band efficiency in SI prior to conscious tactile perception complement each other for optimal processing of weak stimuli. While the original win2con framework^9^,^15^ focused on enhanced integration of task-relevant sensory areas prior to conscious perception, the present study provides important additional insights about spectrally specific network dynamics. As discussed above, the same task-relevant sensory region showed an inverse integration pattern in two frequency bands, suggesting a spectral shift in the connectivity patterns to be essential for the tactile window to consciousness.

### The integration and segregation reflect processes distinct from local excitability

Regarding the relationship of pre-stimulus functional connectivity and power modulations, the integration/segregation effects reported here do not overlap in the time- or the frequency-domains with the pre-stimulus alpha power decreases. In the beta band, the local efficiency decrease was found in the same sensory region as part of the power decreases (SI contralateral to stimulation). While this could reflect a potential power confound, the power and the connectivity effects do not overlap in the time- or frequency-domains. Specifically, whereas the power effect was strongest in high beta shortly before stimulus onset, the local efficiency effect was most pronounced in low beta up to 600 ms pre-stimulus. Regarding the connectivity effects in the theta band, no corresponding power effect was observed. Thus, exceeding previous works, we show network mechanisms in the theta and beta band that reflect most likely additional processes distinct from power modulations in general, and from alpha band power modulations specifically.

This account is particularly well suited to link the pre-stimulus states with well-established theoretical frameworks of conscious perception concerning stimulus-related neural activity. According to the GNW model^14^,^29^, weak sensory input will only be consciously processed if the activity in essential nodes, such as primary sensory areas, is propagated to higher-order areas, causing a global ignition. In contrast, activity constrained to an essential node will not result in a conscious percept^29^. According to this reasoning, decreased local alpha power in a primary sensory area – an essential node with increased cortical excitability – prior to a weak stimulus will not guarantee a downstream spread of neural activity. However, without this propagation to higher-order areas neural activity would quickly fade away, and thus, would not suffice for conscious perception. Our results strongly suggest that this propagation of activity is only possible if relevant functional connections between primary sensory areas and higher-order regions are already contained in the pre-stimulus brain states whereas irrelevant connections are minimized. If this is the case, activity caused by a weak stimulus can be broadcasted effectively, leading to a conscious percept. Thus, as predicted by win2con^9^,^15^, pre-stimulus increased functional integration and segregation provide a plausible explanation for subsequent conscious perception.

## Conclusion

In conclusion, in the present study, we scrutinised our framework win2con^9^ using an NT tactile detection paradigm. We replicate that decreased pre-stimulus alpha power in task-relevant areas is related to the conscious perception of the NT stimulus. Importantly, we show that task-relevant areas are characterised by enhanced and more efficient network integration in the theta band, and less efficient integration in the beta band. In our view, these spectrally specific network patterns indicate a tuning of pre-stimulus pathways by establishing relevant and minimizing irrelevant connections. The resulting pre-stimulus functional pathways then influence how subsequent information can propagate to higher order areas and, therefore, within the global workspace. Taken together, these findings provide evidence for a tactile windows to consciousness characterised by a frequency-specific integration and segregation of the SI contralateral to stimulation into a distributed network.

## Methods

### Participants

19 participants (6 females; mean age: 27 years, SD: 4 years) took part in the experiment after giving written informed consent. All participants were right-handed (Edinburgh Handedness Questionnaire, mean: 95.1, SD: 11.5; 37) and had normal or corrected-to-normal vision. The experimental protocol was approved by the Ethical Committee of the University of Trento, Italy, and the methods were carried out in accordance with the approved guidelines.

### Task and design

To study conscious somatosensory perception, a NT tactile perception task was employed. Tactile stimulation was delivered to the tip of the left index finger, using one finger module of a piezo-electric stimulator (Quaerosys, Schotten, Germany) with 2 × 4 rods, which can be raised to maximally 1 mm. The module was attached to the finger with tape, and the participants’ left hand was cushioned to prevent any unintended pressure on the module. Participants were asked to fixate a black cross on a grey screen throughout the whole experiment to minimise eye movements. To ensure that participants did not hear any auditory cues caused by the piezo-electric stimulator during tactile stimulation, binaural white noise was presented using a STIM2 system (Tip-300, Nicolet, Madison, WI, USA) and MEG-compatible tubal in-ear headphones.

In a training session prior to the main experiment, participants’ individual perceptual threshold was determined in the shielded room using a 1-up/1-down staircase procedure. Two randomly interleaved staircases (one up- and one downward) were used with fixed step sizes. Then, a short training run with 20 trials was conducted to ensure that participants had understood the task, and to control the accuracy of the threshold measurement.

The main experiment consisted of a NT tactile detection task (see Fig. 1A). Participants were told that on each trial a weak tactile stimulus could be presented on the tip of their left index finger at random time intervals. After 250 ms, participants were prompted with an on-screen question to indicate whether they had felt the stimulus (maximal response time: 2 s). Responses were given by using MEG-compatible response boxes with the right index and middle fingers. Overall, there were five to eight runs with 62 trials each. Each trial started with a variable inter-stimulus interval (2–5 s, gamma-distributed) followed by an experimental stimulus (48 per run), a sham stimulus (12 per run) or a catch stimulus (2 per run) of 50 ms each, presented at, clearly below, or clearly above perceptual threshold intensity, respectively. Each run lasted for approximately 5 min; between the runs participants could take a break.

### MEG data acquisition and preprocessing

Electromagnetic brain activity was recorded using a 102 triple-sensor (two planar gradio-, one magnetometer) MEG system (Elekta Neuromag, Helsinki, Finland). Data was sampled continuously at 1 kHz. Prior to the experiment, the headshape of each participant was measured using a Polhemus FASTRAK 3D digitiser, relative to five coils (two on the left and right mastoid, three coils on the front). Head movement was monitored by passing small currents through these coils before each run. From most participants, an anatomical 3D structural image was obtained using a 4T magnetic resonance imaging (MRI) scanner (Bruker Biospin, Ettlingen Germany). All MEG data was analysed using the Matlab-based Fieldtrip toolbox^38^. Epochs of +/−2000 ms length were extracted around stimulus onset and 1 Hz highpass filtered. Then, the data was visually inspected to identify and remove noisy trials, channel jumps and ocular artefacts. After the artefact rejection, all trials were downsampled to 300 Hz. In all further analyses, an equal number of detected and undetected trials was randomly selected to prevent any bias across conditions^39^.

### Sensor-level analyses

Sensor-level analyses were done for both sensor types separately and missing channels were interpolated. Neural activity event-related to stimulus onset was investigated by computing event-related fields (ERF). To this end, 30 Hz lowpass-filtered epochs were averaged, and normalized by subtracting the mean activity in a pre-stimulus baseline time window (−300 to −100 ms). Furthermore, spectral power was estimated using a Fourier transformation on Hanning-tapered time windows from 1500 ms pre- to 500 ms post-stimulus (in steps of 50 ms) from 2 to 30 Hz (in steps of 1 Hz). The length of the sliding time window was frequency dependent (6 cycles per frequency).

### Source-level analyses

For all source-level analyses, the preprocessed data was bandpass-filtered between 2–30 Hz and projected to source-level using an LCMV beamformer analysis^40^. For each participant, realistically shaped, single-shell headmodels^41^ were computed by co-registering the participants’ headshape either with their structural MRI or – when no individual MRI was available (6 participants) – with a standard brain from the Montreal Neurological Institute (MNI, Montreal, Quebec, Canada; http://www.bic.mni.mcgill.ca/brainweb), warped to the individual headshape. A grid with 1.5 cm resolution created based on an MNI template brain was morphed into the brain volume of each participant. A common spatial filter (for each grid point and each participant) was computed using the leadfields and the common covariance matrix taking into account the data from both conditions (detected, undetected). Using this common filter, the spatial power distribution was estimated for the detected and undetected trials separately.

For the source-level event-related activity, the covariance window for the beamformer filter calculation was based on 100 ms pre- to 300 ms post-stimulus. The resulting data was averaged relative to the stimulus onset in both conditions (detected and undetected), and baseline-normalized relative to a time-window from 300-100 ms pre-stimulus. To eliminate polarity, statistics were computed on the absolute values of the source-level event-related responses (ER).

For the source-level analysis of spectral power and connectivity in the pre-stimulus time period, the LCMV beamformer filter was calculated based on a covariance window from 1000–100 ms pre-stimulus. Spectral power was estimated using a multitaper FFT method on dpss-tapered time windows from 1500-0 ms pre-stimulus (in 100 ms steps) for 2–30 Hz (in 2 Hz steps) with a frequency-smoothing of 3 Hz. The length of the sliding time window was frequency dependent (5 cycles per frequency). Furthermore, functional connectivity was calculated using imaginary coherence^42^ and graph theoretical analysis was applied to the thresholded connectivity matrices^27^. Imaginary coherence values were obtained based on the spectral power analysis described above. Then, the absolute values of the resulting coherence spectra were thresholded for each frequency-band and across conditions to obtain binary adjacency matrices. Thresholds were determined by first identifying the smallest of all maximal coherence values per node within each frequency band and each condition. This procedure ensured that each node had at least one connection without underestimating actual connections. Then, the lower threshold of either condition was chosen and applied to both conditions. All coherence values below this were set to zero. For the individual nodes following graph theoretical measures were calculated: node degree (number of connections for one specific node), local efficiency (inverse of average path lengths of all direct connections of a node), local clustering (proportion of connections between direct connections of a node to the total amount of possible connections), and local betweenness (placement on many shortest paths of the network^27^,^28^). While node degree reflects the overall connectedness of a node, local efficiency and clustering is more sensitive to its integration, and local betweenness reflects the importance of a node in the whole network^27^. We also computed global graph theoretical measures such as density, average path length, efficiency, and small-worldedness. However since no effects were obtained that survived correction for multiple comparisons we refrain from a detailed description of these measures for brevity’s sake.

### Statistical testing

Detection rates for the experimental trials were statistically compared to those from the catch trials as well as to chance level, using a dependent samples T-Test. Concerning the MEG data, the main statistical contrast was between trials in which participants reported a stimulus detection, with trials in which they did not (detected vs. undetected). If not stated differently, these two conditions were statistically tested with a dependent-samples T-test, controlling for multiple comparisons with a non-parametric cluster-based permutation analysis^43^.

Event-related activity was tested for a time-window from 0–1000 ms post-stimulus on sensor and source level. Spectral power was tested for a 1000–100 ms pre-stimulus time-window, and the theta, alpha and beta band frequency window (2–6 Hz, 8–14 Hz and 16–26 Hz). On sensor-level, this was done separately for the two sensor types with averaging over frequency-bands. On source-level, based on the sensor-level results, the statistical test was done only for the right hemisphere (contralateral to stimulation) with averaging over the whole time-frequency windows.

Concerning graph theory, for the local measures, the data was averaged across the time-window of 1000-100 ms pre-stimulus, and frequency-windows in the theta, alpha, and beta band (2–6 Hz, 8–14 Hz, 16–26 Hz). The main contrast (detected vs. undetected) was then tested within an anatomically defined region of interests (ROI; the SI contralateral to stimulation) with a dependent-samples T-test using a non-parametric permutation analysis and a false-discovery rate correction for multiple comparisons across voxels (FDR)^44^.

## Additional Information

**How to cite this article**: Frey, J. N. *et al.* The Tactile Window to Consciousness is Characterized by Frequency-Specific Integration and Segregation of the Primary Somatosensory Cortex. *Sci. Rep.* **6**, 20805; doi: 10.1038/srep20805 (2016).
